# Early administration of RS 67333, a specific 5-HT4 receptor agonist, prevents amyloidogenesis and behavioral deficits in the 5XFAD mouse model of Alzheimer’s disease

**DOI:** 10.3389/fnagi.2013.00096

**Published:** 2013-12-24

**Authors:** Patrizia Giannoni, Florence Gaven, Dimitri de Bundel, Kevin Baranger, Evelyne Marchetti-Gauthier, François S. Roman, Emmanuel Valjent, Philippe Marin, Joël Bockaert, Santiago Rivera, Sylvie Claeysen

**Affiliations:** ^1^CNRS, UMR-5203, Institut de Génomique FonctionnelleMontpellier, France; ^2^Inserm, U661Montpellier, France; ^3^Universités de Montpellier 1 and 2, UMR-5203Montpellier, France; ^4^Aix-Marseille Univ, Neurobiologie des Interactions Cellulaires et Neurophysiopathologie, UMR 7259Marseille, France; ^5^CNRS, NICN, Neurobiologie des Interactions Cellulaires et Neurophysiopathologie, UMR 7259Marseille, France; ^6^Service de Neurologie et de Neuropsychologie, CHU La Timone, AP-HMMarseille, France

**Keywords:** alpha-secretase, sAPP alpha, serotonin, amyloid plaques, preventive pharmacotherapy, G protein-coupled receptor

## Abstract

Amyloid β (Aβ) accumulation is considered the main culprit in the pathogenesis of Alzheimer’s disease (AD). Recent studies suggest that decreasing Aβ production at very early stages of AD could be a promising strategy to slow down disease progression. Serotonin 5-HT_4_ receptor activation stimulates α-cleavage of the amyloid precursor protein (APP), leading to the release of the soluble and neurotrophic sAPPα fragment and thus precluding Aβ formation. Using the 5XFAD mouse model of AD that shows accelerated Aβ deposition, we investigated the effect of chronic treatments (treatment onset at different ages and different durations) with the 5-HT_4_ receptor agonist RS 67333 during the asymptomatic phase of the disease. Chronic administration of RS 67333 decreased concomitantly the number of amyloid plaques and the level of Aβ species. Reduction of Aβ levels was accompanied by a striking decrease in hippocampal astrogliosis and microgliosis. RS 67333 also transiently increased sAPPα concentration in the cerebrospinal fluid and brain. Moreover, a specific 5-HT_4_ receptor antagonist (RS 39604) prevented the RS 67333-mediated reduction of the amyloid pathology. Finally, the novel object recognition test deficits of 5XFAD mice were reversed by chronic treatment with RS 67333. Collectively, these results strongly highlight this 5-HT_4_ receptor agonist as a promising disease modifying-agent for AD.

## INTRODUCTION

Alzheimer’s disease (AD) is currently recognized as one of the most socially devastating neurodegenerative disorders (Ferri et al., 2005). AD pathogenesis is complex and relates to the dysfunction of multiple systems. Current treatments to promote acetylcholine (ACh) transmission or to inhibit NMDA receptors produce only symptomatic benefits (Melnikova, 2007), underscoring the urgent need to find disease-modifying treatments.

Alzheimer’s disease neuropathological hallmarks include extracellular deposits of amyloid β (Aβ) peptides (amyloid plaques) and intracellular aggregates of hyper-phosphorylated Tau protein (neurofibrillary tangles; Glenner and Wong, 1984; Kang et al., 1987; Alzheimer et al., 1995). According to the amyloid cascade hypothesis, Aβ peptides and in particular the oligomeric forms are critical determinants of synaptic loss and cognitive deficits in AD (Hardy and Selkoe, 2002). In line with this theory, recent results from Phase III clinical trials indicate that Solanezumab, an anti-Aβ monoclonal antibody, can bring some cognitive benefit to patients with mild AD-related dementia (Aisen et al., 2013), while late interventions were unsuccessful (Sperling et al., 2013). Focusing on prevention, three new studies are enrolling candidates to test anti-amyloid agents in asymptomatic AD patients (Mullard, 2012).

Aβ peptides are generated by sequential β- and γ-secretase cleavage of the amyloid precursor protein (APP). Conversely, non-amyloidogenic cleavage by α-secretase within the Aβ sequence releases the neuroprotective soluble APP (sAPPα) fragment and precludes Aβ generation (Fahrenholz, 2007). Previous reports have shown that G protein-coupled receptors can enhance sAPPα production by stimulating α-secretase activities. They include several neurotransmitter receptors such as muscarinic M_1_–M_3_ acetylcholine receptors [initially demonstrated by Nitsch et al. (1992)], mGlu2 metabotropic glutamate receptor, serotonin 2A (5-HT_2A_), and 2C (5-HT_2C_) receptors [reviewed in Thathiah and De Strooper (2011)]. Activation of serotonin type 4 (5-HT_4_) receptors also stimulates APP α-cleavage and constitutes an increasingly attractive therapeutic strategy against amyloid toxicity. We and others have demonstrated that 5-HT_4_ receptor agonists promote the release of sAPPα *in vitro* and *in vivo* (Maillet et al., 2003; Cochet et al., 2013), an effect that reflects the reduction in Aβ production and deposition (Cho and Hu, 2007; Hashimoto et al., 2012; Tesseur et al., 2013). 5-HT_4_ receptor agonists also improve memory deficits by increasing ACh neurotransmission (Consolo et al., 1994; Bockaert et al., 2011; Johnson et al., 2012). Moreover, Donepezil, an acetylcholinesterase inhibitor, acts in synergy with 5-HT_4_ receptor agonists to enhance sAPPα release and to exert promnesic effects in behavioral tests in mice (Cachard-Chastel et al., 2008; Freret et al., 2012). In addition, a post-mortem study showed a reduction of 5-HT_4_ receptor density in brains of AD patients (Reynolds et al., 1995). Thus, 5-HT_4_ receptor activation may have beneficial effects in AD both by reducing Aβ production and by improving memory performances.

Here, we tested the hypothesis that chronic administration of the 5-HT_4_ receptor agonist RS 67333 may shift APP cleavage toward sAPPα production, thus inhibiting amyloid formation and improving the cognitive performance in 5XFAD mice, a model of AD (Oakley et al., 2006). Our results suggest that this 5-HT_4_ receptor agonist efficiently slows down amyloidogenesis during the prodromal-like stage of the pathology and highlight the importance of early intervention to enhance the chances of significant therapeutic effects.

## MATERIALS AND METHODS

### MICE

Animal experiments were carried out in accordance with the Directive by the Council of the European Communities of November 24, 1986 (86/609/EEC). All efforts were made to minimize animal suffering and to reduce the number of mice used. Wild type (WT) male C57BL/6 mice (8-week-old) were obtained from Janvier (Le Genest-Saint-Isle, France). The generation of 5XFAD mice was described previously (Oakley et al., 2006). These transgenic mice overexpress both human APP (695) harboring the Swedish (K670N, M671L), Florida (I716V) and London (V717I) familial AD (FAD) mutations and human Presenilin 1 (PS1) harboring the two FAD mutations M146L and L286V. Expression of both transgenes is regulated by neuronal-specific elements of the mouse *Thy1 *promoter. The 5XFAD strain (B6/SJL genetic background) was maintained by crossing hemizygous transgenic mice with B6/SJL F1 breeders (Janvier). 5XFAD heterozygous transgenic mice were used for the experiments and WT littermates as controls. All animals were genotyped by PCR using tail genomic DNA. Transgenic and WT mice were bred in our animal facility, had access to food and water *ad libitum* and were housed under a 12 h light-dark cycle at 22–24°C. Female 5XFAD mice were used in this study. For each experiment, the age of mice is indicated in months (with a 2-week range).

### DRUGS

The following compounds were used: RS 67333 (1-(4-amino-5-chloro-2-methoxy-phenyl)-3-(1-butyl-4-piperidinyl)-1-propa-none) and RS 39604 (1-[4-amino-5-chloro-2-(3,5-dimethoxy-benzyl-oxy)phenyl]-3-[1-[2-[(methylsulfonyl)amino]ethyl]]-4-piperidinyl]]-1-propanone hydrochloride). All drugs were purchased from Tocris Bioscience (R&D Systems Europe, Lille, France).

### ANIMAL TREATMENTS

To induce acute 5-HT_4_ receptor activation in non-pathological conditions, four different groups of WT C57BL/6 mice (*n* = 6/group) received one intraperitoneal (i.p.) injection of vehicle (0.9% w/v NaCl; 0.2% dimethyl sulfoxide in water), RS 67333 (selective 5-HT_4_ receptor partial agonist), RS 39604 (a 5-HT_4_ receptor antagonist), or both drugs (the antagonist was administered 15 min before the agonist; 1 mg/kg each drug). Animals were sacrificed 30 min after the injection, heads were quickly frozen in liquid nitrogen, skulls opened on ice, and frontal cortex and hippocampus dissected and stored at -80°C for further analysis.

5XFAD mice and WT littermates received chronic treatments with drugs or vehicle according to three different protocols. In each experiment, drugs or vehicle solution were administered i.p. twice a week (1 mg/kg). In “Protocol 1,” mice (*n* = 5) were treated with RS 67333 from 1 to 4 months of age. In “Protocol 2,” mice (*n* = 6) were treated for 2 months, from 2 to 4 months of age. In “Protocol 3,” RS 67333 was administered for 1 month, from 2 to 3 months of age (*n* = 8). In addition, to antagonize RS 67333 effects, four different groups of mice (*n* = 4 each) received vehicle, RS 67333, RS 39604, or both drugs (the antagonist was administered 15 min before the agonist) according to protocol 2. At the end of each protocol, mice were anesthetized with a mixture of 100 mg/kg ketamine and 10 mg/kg xylazine in saline solution and perfused transcardially with PBS. Brains were quickly isolated on ice, the olfactory bulbs and cerebellum removed and the two hemispheres divided. One hemisphere was frozen on dry ice and stored at -80°C for biochemical analysis, while the other was post-fixed in 4% PFA for immunohistochemistry (IHC). WT mice, which do not develop plaques, were used to investigate the possible toxic effects of the drugs.

### DETERMINATION OF sAPP PRODUCTION IN TRANSFECTED CELLS

COS-7 cells were grown in Dulbecco’s modified Eagle medium (DMEM) supplemented with 10% dialyzed fetal calf serum (dFCS) and antibiotics. Cells were transfected with plasmids encoding HA-tagged 5-HT_4_ receptor and secreted placental alkaline phosphatase (SEAP)-tagged mouse APP695, as previously described (Cochet et al., 2013), and then seeded in 24-well plates (250,000 cells/well). Twenty-four hours after transfection, cells were incubated with the appropriate drug concentration for 30 min, then culture supernatants were collected and SEAP activity measured by adding the chromogenic substrate *p*-Nitrophenyl phosphate disodium hexahydrate (Sigma-Aldrich, Saint-Quentin Fallavier, France) according to the manufacturer’s instructions. The reaction readout was performed at 405 nm using an Infinite 2000 luminescence counter (Tecan, Männedorf, Switzerland).This technique measures all secreted soluble forms of APP and does not discriminate between sAPPα and sAPPβ.

### CEREBROSPINAL FLUID COLLECTION

Mice were anesthetized and mounted onto a stereotaxic instrument. The neck skin was cut and subcutaneous tissue and muscles separated with the help of micro-retractors (Fine Science Tools, Heidelberg, Germany). Mice were then laid down so that the head formed an angle of about 135° with the body (Liu and Duff, 2008). A capillary tube (Borosilicate glass, B100-75-10, Sutter Instruments, Novato, CA, USA) was used to punch the *dura mater* of the *cisterna magna.* Cerebrospinal fluid (CSF) was collected by capillary action and transferred to 0.5 mL microtubes, immediately frozen on dry ice and stored at -80°C until use. Once thawed, samples were heated at 60°C for 5 min as described in Bourdin et al. (2008) and analyzed without further freezing-thawing cycles.

### BRAIN EXTRACT PREPARATION

Brain hemispheres, frontal cortex and hippocampus of 5XFAD mice and controls were thawed, weighed, and homogenized in four volumes of tris-saline (50 mM Tris-HCl pH = 7.4, 150 mM NaCl) with a protease inhibitor cocktail (Roche Applied Science, Meylan, France). The resulting homogenates were centrifuged at 540,000 × *g* for 20 min and supernatants (the “soluble fraction”) collected and aliquoted for storage at -80°C. Pellets were resuspended by brief sonication in 10 volumes of 6 M guanidine HCl in 50 mM Tris-HCl, pH = 7.6 and centrifuged again at 265,000 × *g* for 20 min. Supernatants (the “insoluble fraction”) were aliquoted and stored at -80°C (Morishima-Kawashima et al., 2000).

### QUANTIFICATION OF Aβ40, Aβ42, and sAPPα

ELISA kits from IBL International (Hamburg, Germany) for the dosage of Aβ_40_ [human amyloid β (1–40) assay kit, #27713], Aβ_42_ [human amyloid β (1–42) assay kit, #27719] or sAPPα (mouse/rat sAPPα assay kit, #27415; human sAPPα assay kit, #27734) were used according to the manufacturer’s instructions. Reactions were read at 620 and 450 nm using an Infinite 2000 luminescence counter. The obtained values were normalized to the protein concentration of each sample, measured using a BCA protein assay (Sigma-Aldrich). The sAPPα ELISA kits enable the precise and selective quantification of sAPPα versus sAPPβ.

### IMMUNOHISTOCHEMISTRY

Thirty-micrometer-thick sections were cut using a vibratome (Microm HM 650 V, Thermo Scientific, Saint Herblain, France) and stored in cryoprotectant medium at -20°C. For the labeling of amyloid plaques, free-floating tissue sections of frontal cortex, hippocampus, and entorhinal cortex (coordinates from the bregma: frontal cortex = 1.98 mm, hippocampus = -1.94 mm, entorhinal cortex = -3.08 mm) were extensively washed in PBS and then incubated in blocking solution (PBS; 3% BSA; 0.1% Triton X-100) for 1 h. Sections were stained with Hoechst dye (1:1000, Life Technologies, Saint Aubin, France) for 15 min to detect cell nuclei and then with freshly prepared thioflavin T solution (#T3516-5G, Sigma-Aldrich; final concentration: 0.01 mg/ml in blocking buffer) for 15 min. After washing in 70% ethanol for 5 min, samples were mounted on poly-lysine slides with coverslips. For GFAP (glial fibrillary acidic protein) or IBA1 (ionized calcium-binding adapter molecule 1) staining, free-floating brain sections were blocked as before and incubated with polyclonal rabbit anti-GFAP (1:1000, Z0334, Dako, Les Ullis, France) or anti-IBA1 antibodies (1:3000, 019-19741, Wako Chemicals GmbH, Neuss, Germany) at 4°C overnight. After thioflavin T staining and washing with 70% ethanol, the secondary Alexafluor 594 goat anti-rabbit antibody (1:1000, A11012, Life Technologies) was added for 2 h. PBS washes and mounting were performed as described before.

### IMAGE ACQUISITION AND ANALYSIS

Images were acquired with an AxioImager Z1 microscope (Carl Zeiss S.A.S., Marly le Roi, France). Analysis of thioflavin T staining was performed blindly and data are presented as the mean number of particles per mm^2^ in two tissue sections from the same brain area/animal (Image J software). GFAP and IBA1 expression were quantified in hippocampus (*dentate gyrus*) using the same method and results were expressed as area fractions. For representative images of thioflavin T and GFAP staining, mosaic sequential scans with a 10× objective were taken. Detailed images of plaques were captured with a 40× objective and selected *Z*-stacks were pooled together.

### NOVEL OBJECT RECOGNITION TEST

The cognitive performance of mice was tested using the novel object recognition (NOR) test (Bevins and Besheer, 2006). Animals were extensively handled during drug treatment prior to the test onset. Each day, mice were allowed to familiarize with the test room for at least 1 h prior to the test. Testing was carried out in a Plexiglas box (width: 35 cm, length: 20 cm, height: 20 cm) placed in a dimly lit room. On day 1 and 2, each mouse was habituated to the empty box for 10 min/day. On day 3, two objects (constructed out of plastic toys) were positioned in the cage, 5 cm away from the opposing walls. During the training session, each animal was placed between the two objects, facing the wall and then was allowed to explore the objects for 5 min. Mice were then returned to their home cage and 1 h later went through a 5 min test session in which one of the two (familiar) objects was replaced by a new one (novel). The whole experiment was video-recorded and object exploration (time spent by the mouse nose in contact with the object or by sniffing it at a distance ≤ 1 cm) was blindly measured. Two parameters were considered: (1) the exploration time (s) spent by the animal interacting with the two familiar objects during the training session and (2) the exploration time spent by the animal interacting with the novel object relative to the total exploration time {[novel/(familiar + novel)] × 100} during the test. A discrimination index was also calculated {[novel - familiar]/[familiar + novel]}. Mice that failed to explore either of the two objects or explored both objects for less than 10 s during the training session were excluded from the data analysis (two WT and two 5XFAD mice from a total of 28 mice).

### STATISTICAL ANALYSIS

All values are expressed as the mean ± SEM. Significant effects of treatments were determined by ANOVA analysis followed by Bonferroni’s or Tukey’s *post hoc* tests in the case of multiple comparison groups. In all other cases, the unpaired Student’s *t* test was used. For all statistical tests, *p *< 0.05 was considered significant. Analysis was performed with GraphPad Prism 6.0a (GraphPad Software, La Jolla, CA, USA).

## RESULTS

### 5-HT_4_ RECEPTOR AGONISTS INDUCE sAPPα RELEASE *IN VITRO* AND *IN VIVO*

**FIGURE 1:**
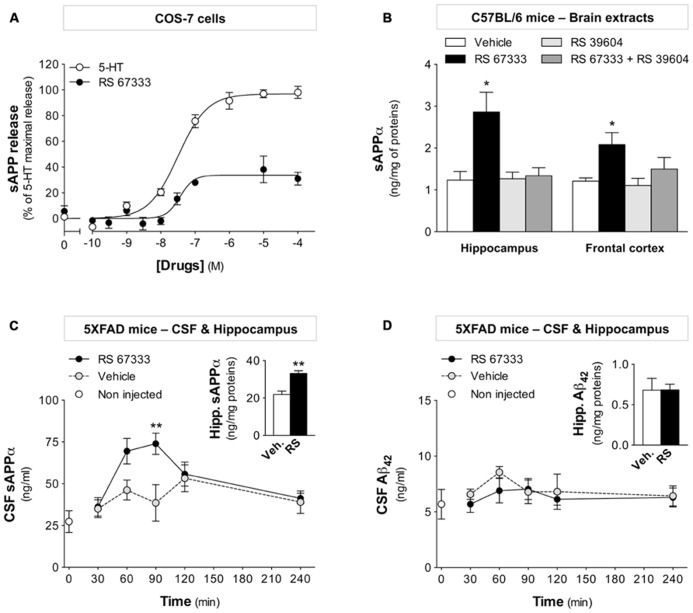
**The RS 67333 5-HT_4_ receptor agonist induces sAPPα release *in vitro* and *in vivo*.** **(A)** COS-7 cells that transiently express 5-HT_4_ receptors and SEAP-APP were stimulated with increasing concentrations of serotonin or RS 67333 for 30 min and then sAPP release in culture supernatants was measured by quantification of alkaline phosphatase activity. Data are the mean ± SEM of values obtained in a typical experiment performed in triplicate. Two other experiments performed on different sets of cultured cells yielded similar results. **(B)** sAPPα levels in hippocampus and frontal cortex extracts of C57BL/6 mice were quantified 30 min after one i.p. injection of RS 67333 (1 mg/kg) and/or of the 5-HT_4_ receptor antagonist RS 39604 (1 mg/kg, 15 min before RS 67333). sAPPα values are expressed in nanogram per milligram total proteins (*n* = 6/group). **(C,D)** Kinetics analysis of sAPPα release **(C)** and Aβ_42_ concentration **(D)** in CSF samples from 5XFAD mice after acute RS 67333 administration (1 mg/kg, i.p., *n* = 5/group). Insets show sAPPα **(C)** and Aβ_42_ **(D)** quantification in hippocampal brain extracts at the 30 min endpoint. Data are the mean ± SEM. ^*^p < 0.05, ^**^p < 0.01 compared with vehicle (unpaired Student’s *t* test and two-way ANOVA followed by Bonferroni’s test).

To study sAPPα release upon acute 5-HT_4_ receptor activation in non-pathological conditions, COS-7 cells that transiently express 5-HT_4_ receptors and SEAP-tagged APP were stimulated with increasing concentrations of the highly selective and widely characterized 5-HT_4_ receptor agonist RS 67333 (Eglen et al., 1995; Marchetti et al., 2000; Lamirault and Simon, 2001; Kemp and Manahan-Vaughan, 2004) or with the natural agonist serotonin (5-HT) for 30 min. Quantification of sAPP in culture supernatants by detection of alkaline phosphatase activity, as previously described (Cochet et al., 2013), showed that both treatments increased sAPP release and that 5-HT had the most prominent effect (*E*_max_ = 34 ± 5% of 5-HT maximal release for RS 67333; **Figure 1A**). Then, the release of sAPPα was investigated *in vivo* in WT C57BL/6 mice after a single administration of RS 67333 (1 mg/kg, i.p.), as previously done for the two 5-HT_4_ receptor agonists prucalopride and ML 103022 (Cachard-Chastel et al., 2007). Thirty minutes after the injection, mice were sacrificed and sAPPα concentration was measured in brain extracts from the frontal cortex and hippocampus, two regions that express 5-HT_4_ receptors. Upon 5-HT_4_ receptor activation, sAPPα level increased 2.33-fold (hippocampus) and 1.73-fold (frontal cortex) relative to control values (**Figure 1B**). RS 67333-mediated sAPPα release in both brain regions was prevented when the specific 5-HT_4_ receptor antagonist RS 39604 (Hegde et al., 1995) was injected (1 mg/kg, i.p.) before RS 67333. On its own, RS 39604 did not have any significant effect (**Figure 1B**). Finally, acute 5-HT_4_ receptor activation by RS 67333 (one i.p. injection, 1 mg/kg) increased significantly sAPPα release also in the 5XFAD transgenic mouse model of AD). Specifically, sAPPα concentration in CSF from treated 5XFAD mice reached a peak 90 min post-injection [(sAPPα) = 38.6 ± 10.9 for vehicle- and 73.9 ± 6.3 ng/ml for RS 67333-treated mice; *n* = 5/group] and returned to basal levels 4 h after drug delivery (**Figure 1C**). sAPPα level was also significantly higher (1.51-fold relative to control values) in the hippocampus of RS 67333-treated 5XFAD mice in comparison to controls (**Figure 1C** inset, 30 min post-injection). Conversely, Aβ_42_ concentration in CSF and hippocampus of RS 67333-treated mice was not significantly different compared to controls (**Figure 1D**), indicating that acute 5-HT_4_ receptor stimulation increases sAPPα release in hippocampus and CSF, without affecting Aβ levels in CSF during the 4 h following RS 67333 administration.

### EARLY CHRONIC ADMINISTRATION OF RS 67333 REDUCES THE AMYLOID PLAQUE LOAD IN 5XFAD MICE

**FIGURE 2:**
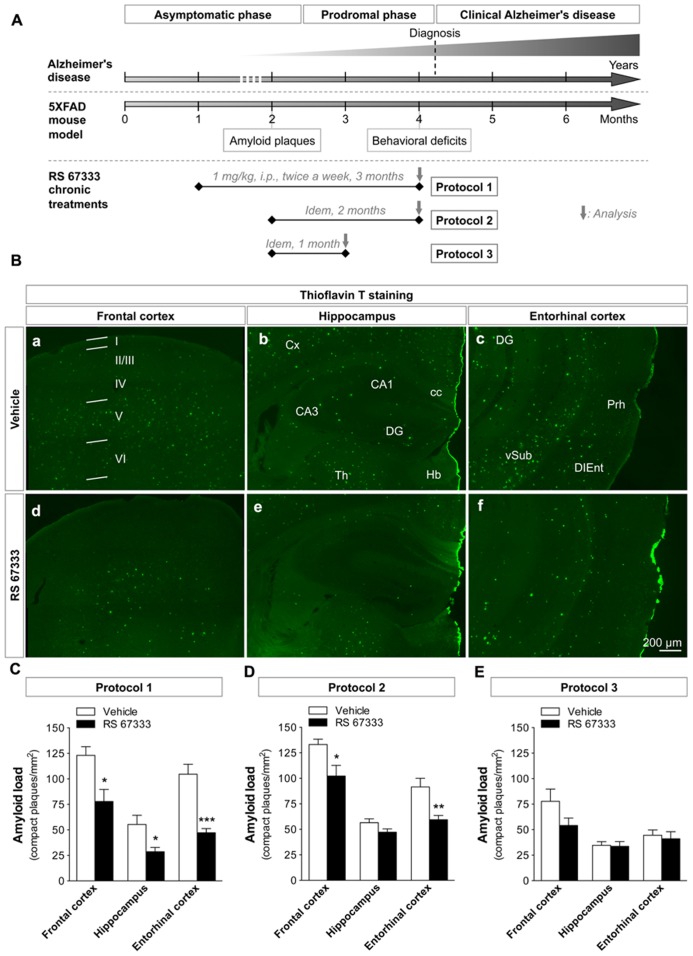
**Early, chronic administration of RS 67333 reduces the amyloid plaque load in 5XFAD mice.** **(A)** Schematic model comparing the progression of AD in humans and of the AD-like pathology in 5XFAD mice. Aβ accumulation in 5XFAD mice starts at 2 months of age, which corresponds to AD asymptomatic phase in humans. The first memory and behavioral deficits in 5XFAD mice appear at about 4 months of age, which corresponds to the prodromal phase preceding the time of diagnosis in humans (clinical AD), prior to progressive accumulation of neuropathological alterations and memory deficits in 5XFAD mice. The time-course of the three different treatment protocols (protocols 1–3) with the 5-HT_4_ receptor agonist RS 67333 (1 mg/kg, i.p., twice a week) is shown below the schematic of disease progression. At the end of the treatment, the behavioral NOR test (only for mice treated according to protocol 2), Aβ_40_ and Aβ_42_ measurements in brain extracts, quantification of amyloid plaques and glial reactivity in brain slices were performed. **(B)** Representative images of thioflavin T staining of amyloid plaques in the frontal cortex (a,d), hippocampus (b,e), and entorhinal cortex (c,f) **(B)** from 5XFAD mice treated with RS 67333 (protocol 1) or vehicle alone. Images are mosaics collected with an AxioImager Z1 microscope, 10X objective (b–d). Quantification of Aβ load (number of plaques/mm^2^) in frontal cortex, hippocampus and entorhinal cortex following RS 67333 treatment using protocol 1 **(C)**, protocol 2 **(D)** or protocol 3 **(E)**. Data are the mean ± SEM, *n* ≤ 5/group. ^*^p < 0.05, ^**^p < 0.01, ^***^p < 0.001 compared with vehicle (unpaired Student’s *t* test). I-VI, cortical layers; Cx, cortex; cc; corpus callosum; CA1 and 3, cornu ammonis areas 1 and 3, DG; dentate gyrus; Th, thalamus; Hb, habenula; Prh, perirhinal cortex; DIEnt, dorsal intermediate entorhinal field; vSub, ventral subiculum.

Amyloid plaques can be already observed in the brain of 2-month-old 5XFAD mice and altered cognitive performances can be detected at 4 months of age (Oakley et al., 2006; Girard et al., 2013). Relative to AD progression in humans, we speculated that 4-month-old 5XFAD mice were entering the “clinical stage of AD” after a prodromal phase and a latent phase of variable duration (**Figure 2A**). To test whether chronic treatment with the 5-HT_4_ receptor agonist RS 67333 could slow down amyloid plaque deposition, we designed three treatment protocols (protocols 1–3) that differed in terms of age at the onset of treatment and duration of treatment, but not in the amount and frequency of administration (1 mg/ kg, i.p., twice a week, in all three; **Figure 2A**). Controls were 5XFAD mice treated with vehicle. Frontal cortex, hippocampus, and entorhinal cortex were analyzed as representative areas of 5XFAD mouse brain (frontal, median, and caudal sections) and of human brain regions that are affected early by AD and are highly enriched in amyloid deposits (Braak and Braak, 1991; Rowe and Villemagne, 2013).

RS 67333 treatment (protocols 1 and 2) strongly decreased the number of amyloid plaques compared to vehicle. The most significant effects were observed with protocol 1 (3 month-treatment, from 1 to 4 months of age, **Figure 2B**), with a drastic reduction of amyloid plaque density (Aβ load) in all the analyzed brain areas (**Figure 2C**; reduction of 37 ± 10% in the frontal cortex, of 48 ± 8% in the hippocampus and of 55 ± 4% in the entorhinal cortex, relative to controls treated with vehicle). In protocol 2 (delayed onset of the treatment, **Figure 2A**), a significant reduction of the plaque number was still observed in the frontal and entorhinal cortices (23 ± 8 and 35 ± 5%, respectively, relative to control), but the small decrease in the hippocampus was not significant (**Figure 2D**). Further shortening of the treatment (1 month, protocol 3, **Figure 2A**), only resulted in a non-significant trend toward a decrease of the plaque number (**Figure 2E**). This suggests that both treatment onset at early age and treatment duration are crucial to obtain robust therapeutic effects.

### CHRONIC ADMINISTRATION OF RS 67333 REDUCES Aβ_40_ and Aβ_42_ LEVELS IN 5XFAD MICE

**FIGURE 3:**
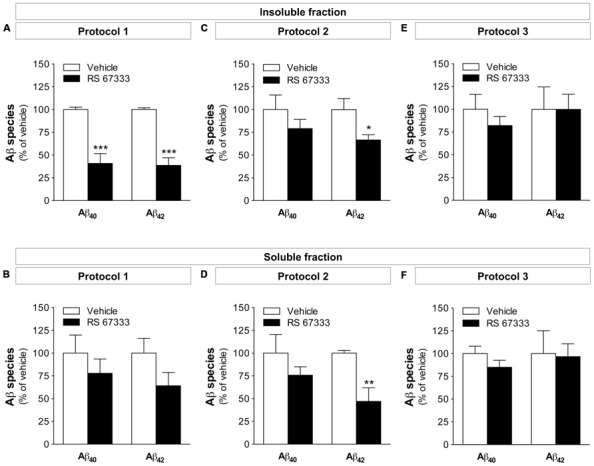
**Early chronic administration of RS 67333 reduces Aβ_40_ and Aβ_42_ levels in 5XFAD mice.** 5XFAD female (3 or 4 months at termination) mice were treated chronically with 1 mg/kg RS 67333 twice a week according to protocol 1 **(A,B)**, protocol 2 **(C,D)** or protocol 3 **(E,F)**. Total Aβ_40_ and Aβ_42_species were quantified in the insoluble **(A,C,E)** and soluble **(B,D,F)** brain fractions. Data are the mean ± SEM. ^*^p < 0.05, ^**^p < 0.01, ^***^p < 0.001 compared with vehicle (unpaired Student’s *t* test). The mean levels (nanogram per milligram total protein ± SEM, *n* = 5/group) in the three vehicle-treated groups (protocol 1, 2, and 3, respectively) were: insoluble Aβ_40_: 483 ± 13, 346 ± 62, and 187 ± 31; insoluble Aβ_42_: 2006 ± 38; 2190 ± 265, and 1477 ± 322; soluble Aβ_40_: 7.62 ± 1.52, 3.97 ± 0.81, and 10.75 ± 0.87; soluble Aβ_42_: 0.44 ± 0.07, 0.82 ± 0.02, and 2.48 ± 0.59.

Consistent with the reduced amyloid plaque load, quantification by ELISA showed a clear reduction of both Aβ_40_ and Aβ_42_ in the insoluble fraction of brain samples from 5XFAD mice treated with RS 67333 following protocol 1 (59 ± 11 and 61 ± 8% reduction of Aβ_40_ and Aβ_42_, respectively, compared to controls; **Figure 3A**). Conversely, Aβ_40_ and Aβ_42_ decrease in the soluble fraction was not significant (**Figure 3B**). In the group treated following protocol 2, only Aβ_42_ levels were significantly reduced in the insoluble and soluble factions (33 ± 6 and 53 ± 15% reduction, respectively, compared to controls; **Figures 3C,D**). One-month treatment with RS 67333 (protocol 3) was not sufficient to affect Aβ_40_ and Aβ_42_ accumulation in brains of 5XFAD mice (**Figures 3E,F**). Collectively, these findings suggest that RS 67333 treatment might impact total brain Aβ content.

### RS 67333-INDUCED REDUCTION OF THE AMYLOID PATHOLOGY IS MEDIATED BY 5-HT_4_ RECEPTOR ACTIVATION

**FIGURE 4:**
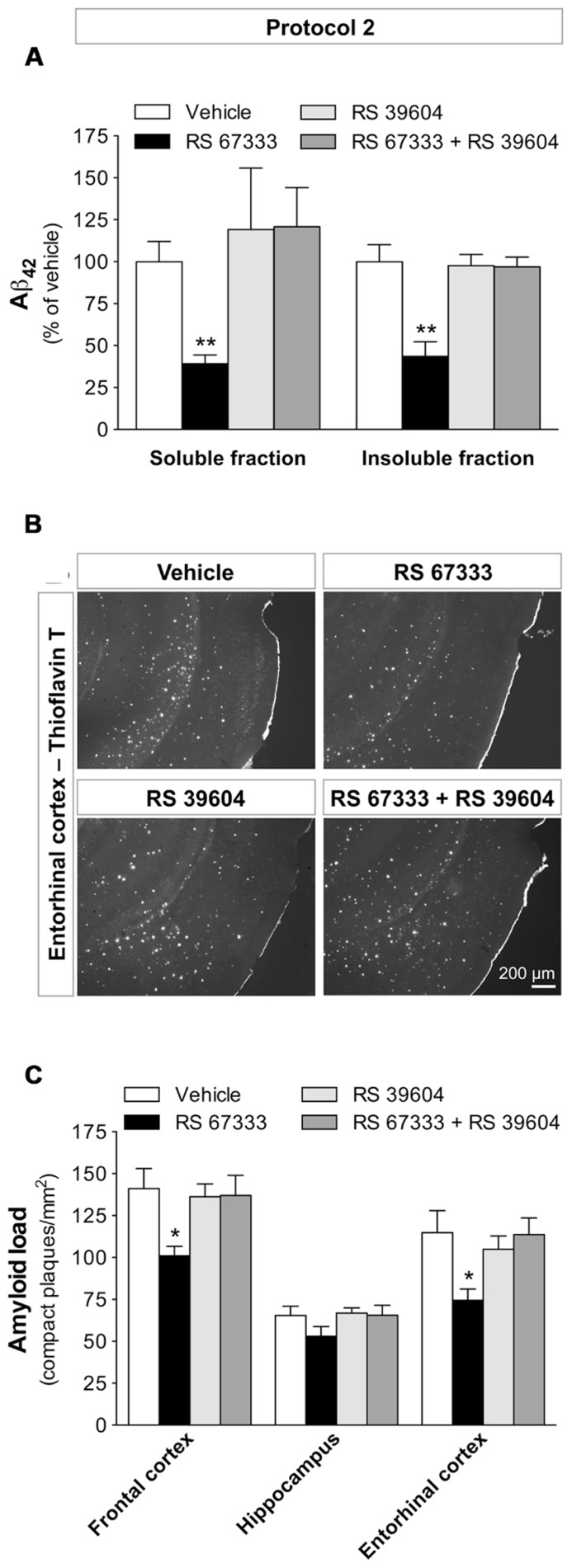
**RS 67333-induced reduction of amyloid pathology is prevented by pre-administration of the 5-HT_4_ receptor antagonist RS 39604.** Two-month-old 5XFAD females were treated with RS 67333 according to protocol 2 (1 mg/kg, twice a week, for 2 months) with or without pre-administration (15 min before) of the 5-HT_4_ receptor antagonist RS 39604. **(A)** Quantification of Aβ_42_ load in the brain insoluble and soluble fractions. The mean concentration (nanogram per milligram total protein ± SEM, *n* = 4/group) of Aβ_42_in the soluble and insoluble fractions from the vehicle-treated group was 0.77 ± 0.10 and 2079.0 ± 203.8, respectively. **(B)**. Representative images of thioflavin T staining in the entorhinal cortex (mosaics, 10× objective). **(C)** Quantification of plaque number. Data are the mean ± SEM. ^*^p < 0.05, ^**^p < 0.01 compared with vehicle (unpaired Student’s *t* test).

To demonstrate the involvement of 5-HT_4_ receptors in the *in vivo* effect of RS 67333, we examined whether RS 39604, a specific 5-HT_4_ receptor antagonist inhibits the protective effect of the agonist in one of the protocols used in our study (protocol 2). Pre-treatment with RS 39604 (administered 15 min before each RS 67333 injection) of a group of 5XFAD mice treated for 2 months prevented the RS 67333-induced reduction in Aβ_42_ levels (**Figure 4A**) and the decrease in plaque formation in the entorhinal (**Figures 4B,C**) and frontal cortices (**Figure 4C**). The 5-HT_4_ receptor antagonist had no effect on the amyloid burden when administered alone for 2 months (**Figures 4A–C**). Collectively, these data demonstrate that 5-HT_4_ receptor activation during the prodromal phase of disease reduces Aβ accumulation and amyloid plaque load in 5XFAD mice.

### CHRONIC ADMINISTRATION OF RS 67333 REDUCES BRAIN TISSUE INFLAMMATION IN 5XFAD MICE

**FIGURE 5:**
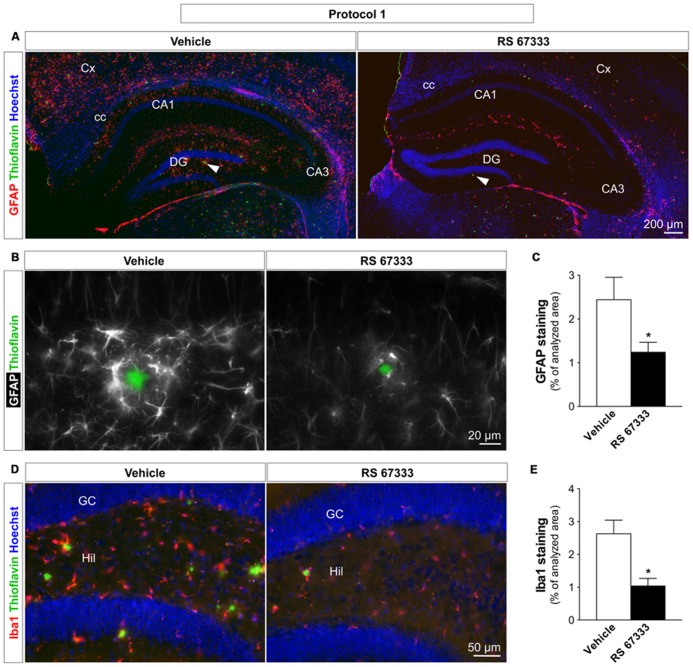
**Early chronic administration of RS 67333 reduces astrogliosis and microgliosis in 5XFAD mice.** One-month-old 5XFAD females were treated according to protocol 1 (1 mg/kg RS 67333 twice a week for 3 months). Representative images of hippocampus sections from controls (vehicle) and treated mice show GFAP [**(A,B)** in red and pseudo-colored in white, respectively], IBA1 [**(D)** in red], thioflavin T [**(A,B,D)** in green] and Hoechst staining [**(A,D)** in blue]. Images in A are mosaics, 10× objective; images in **(B)** are higher magnifications (40× objective) of distinct plaques indicated by white arrowheads in **(A)**. Quantification of GFAP **(C)** and IBA1 **(E)** staining, markers of astroglia and microglia, respectively. Data are presented as the mean ± SEM. ^*^p < 0.05 compared with vehicle (unpaired Student’s *t* test). GFAP, glial fibrillary acidic protein; IBA1, ionized calcium-binding adapter molecule 1; Cx, cortex; cc, corpus callosum; CA1 and 3, cornu ammonis areas 1 and 3; DG, dentate gyrus; GC, granule cells; Hil, hilus.

Increasing evidence indicates that plaque deposition induces astrogliosis and microgliosis in the brain of patients with AD. Over the years, these chronic inflammation processes are likely to contribute to AD progression (Akiyama et al., 2000; Wyss-Coray and Rogers, 2012). We thus asked whether the anti-amyloidogenic effects induced by RS 67333 could also lead to reduction of brain inflammation in 5XFAD mice. Immunohistochemical assessment of astroglial (anti-GFAP antibody) and microglial (anti-IBA1 antibody) activation showed that RS 67333 chronic treatment (protocol 1) strikingly reduced both astrogliosis (49 ± 9% decrease of GFAP staining in hippocampal brain slices of treated animals in comparison to controls that received vehicle; **Figures 5A–C**) and microgliosis (57 ± 9% reduction of IBA1 staining in treated animals compared to controls; **Figures 5D,E**). In protocol 2, a slight but non-significant reduction of astroglial staining was seen in RS 67333-treated 5XFAD mice (data not shown).

### CHRONIC ADMINISTRATION OF RS 67333 REVERSES COGNITIVE DEFICITS IN 5XFAD MICE

**FIGURE 6:**
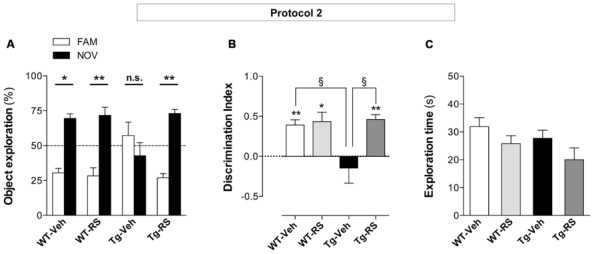
**Chronic administration of RS 67333 prevents object recognition memory loss in 5XFAD mice.** Two-month-old 5XFAD females (Tg) and WT littermates were treated with RS 67333 or vehicle according to protocol 2 (1 mg/kg, twice a week for 2 months) and then recognition memory was assessed with the novel object recognition (NOR) test. Habituation sessions were started 3 days after the end of treatment and the interval between training session and test was of 1 h. **(A)** RS 67333-treatment reverses cognitive impairment as indicated by the higher percentage of time devoted to the exploration of the novel object in comparison to mice treated with vehicle. Data are the mean ± SEM (*n* = 6–7 mice/group). ^*^p < 0.05, ^**^p < 0.01 versus familiar object (two-way ANOVA followed by Bonferroni’s test). **(B)** This finding was confirmed also by calculating the discrimination index [(novel - familiar)/(familiar + novel)]. Data are the mean ± SEM. Indices different from zero: ^*^p < 0.05, ^**^p < 0.01 (unpaired Student’s *t* test). ^§^*p* < 0.05 versus Tg-vehicle group (two-way ANOVA followed by Tukey’s test). **(C)** The total exploration time was not significantly different among groups.

As acute stimulation of 5-HT_4_ receptors exerts pro-cognitive effects on learning and memory (Letty et al., 1997; Marchetti-Gauthier et al., 1997; Freret et al., 2012), we then investigated whether chronic stimulation with RS 67333 (protocol 2, **Figure 2A**) could also improve cognitive performances *via* preventive reduction of Aβ formation. These behavioral studies were performed using protocol 2, which is the most relevant protocol for translation into a treatment of the human pathology (drug delivery at the adult stage). 5XFAD mice and WT littermates were thus treated for 2 months with RS 67333 or vehicle and then their episodic-like memory was tested using the NOR test that is classically employed in mouse models of AD (Bevins and Besheer, 2006). Training sessions were started 3 days after the end of treatment, thus acute effects of RS 67333 cannot influence the test. Body weight and global health monitoring throughout the treatment showed that chronic RS 67333 treatment did not induce adverse effects in 5XFAD and WT littermates. Impaired ability to discriminate between the familiar and novel objects after 1 h retention interval was observed in 5XFAD mice treated with vehicle compared to WT littermates (**Figures 6A,B**), in agreement with the fact that 5XFAD mice start to show altered cognitive functions at 4 months of age (Oakley et al., 2006; Jawhar et al., 2012; Girard et al., 2013). Conversely, cognitive impairment was completely prevented in RS 67333-treated 5XFAD mice (**Figures 6A,B**). RS 67333 did not have a pro-cognitive effect in WT littermates in these experimental conditions. Finally, the total exploration time during the training session did not differ significantly in the four groups (**Figure 6C**), indicating that the recognition memory improvement in 5XFAD mice treated with RS 67333 is not secondary to an increase of the exploratory activity.

## DISCUSSION

In this study, we show that early, chronic administration of the 5-HT_4_ receptor agonist RS 67333 reduces the amyloid burden and inflammation markers (astrogliosis and microgliosis) and prevents memory impairment in 5XFAD mice. We also demonstrate that starting the treatment early (during the prodromal phase of the neurodegenerative disease) and its duration are both crucial to significantly reduce the levels of Aβ species and plaque formation.

Amyloid precursor protein non-amyloidogenic processing and sAPPα release upon agonist-mediated 5-HT_4_ receptor activation is now well established in cell lines and primary neuronal cultures (Maillet et al., 2003; Hashimoto et al., 2012; Cochet et al., 2013; Tesseur et al., 2013). Moreover, both short and chronic (once a day for 10 days) 5-HT_4_ stimulation with high RS 67333 dose (3 mg/kg, i.p.) can reduce amyloidogenesis in 10- to 12-month-old Tg2576 mice (another mouse model of AD) possibly through matrix metalloproteinase-9 (MMP-9) up-regulation (Hashimoto et al., 2012). Another recent study evaluated the effects of acute and chronic treatment with a novel but not fully characterized 5-HT_4_ agonist (SSP-002392) in APP/PS1 mice that express human APP with the Swedish mutations and mutant human PS1 (Tesseur et al., 2013). Despite no evidence for direct α-secretase stimulation in the brain, the authors showed a reduction in soluble and insoluble Aβ in the hippocampus accompanied by a decrease in APP and BACE1 (β-secretase 1/beta-site APP cleaving enzyme 1) expression and elevated astroglial and microglial responses. Moreover, reduction of the amyloid pathology upon chronic treatment (37 days) was detected only in the brain of 4- to 5-month-old but not of 12-month-old APP/PS1 mice, in which abundant Aβ deposits were still present. This previous study is not directly comparable to the current report for the two following reasons: (1) higher and more frequent 5-HT_4_ agonist administration were performed in the previous study; (2) the model used (Tg2576) was far less aggressive and easier to cure than the mouse model that was used here (5XFAD). Therefore, our work brings new insights into the 5-HT_4_ receptor-induced non-amyloidogenic cleavage of APP and the potential of 5-HT_4_ agonists to slow down disease progression.

### TRANSIENT INCREASE OF sAPPα IN THE CSF OF 5XFAD MICE UPON RS 67333 5-HT_4_ AGONIST INJECTION

We observed a significant increase of sAPPα release in both hippocampus and frontal cortex of WT C57BL/6 mice after one i.p. injection of the 5-HT_4_ receptor agonist RS 67333 at low doses (1 mg/kg). This effect was reversed by a 5-HT_4_ receptor antagonist. Moreover, we show for the first time an increase in sAPPα accumulation in the CSF of 5XFAD mice following RS 67333 administration. Kinetic studies indicated that sAPPα was only transiently increased in CSF, a finding that might explain why sAPPα production is difficult to detect in brain samples (Cachard-Chastel et al., 2007; Tesseur et al., 2013)*.* Therefore, to accurately monitor sAPPα level variations in CSF, it might be necessary to precisely identify the optimal sample collection time, based on the drug administration route and bioavailability. This transient sAPPα increase might also explain why we could not detect any increase in sAPPα in the soluble fraction of brain extracts after chronic treatments (not shown). The extent of 5-HT_4_ receptor-mediated sAPPα release in CSF could also constitute a biomarker to follow the capacity to induce non-amyloidogenic APP cleavage in AD brains in which the 5-HT_4_ receptor density is known to be reduced (Reynolds et al., 1995).

### REDUCTION OF Aβ SPECIES AND AMYLOID PLAQUES

The 5-HT_4_ receptor-induced APP α-cleavage should, *de facto*, preclude Aβ formation. Although acute RS 67333 administration did not induce any significant change in the Aβ_42_CSF content within a 4 h-period, we observed a decrease of Aβ_40-42_production and Aβ deposits (plaques) in all brain regions after early, chronic treatment with RS 67333 (up to 3 months). These data are in agreement with previous *in vitro* results showing that RS 67333 inhibits in a dose-dependent manner the generation of Aβ_40__-__42_ species in primary cortical cultures of Tg2576 mice over a 2-day accumulation period (Cho and Hu, 2007).

Amyloid plaques seem to be the final response of the organism to the progressive increase in Aβ concentration (Gandy, 2011). Interestingly, in RS 67333-treated mice, the reduction of insoluble Aβ species was more pronounced than that of soluble fractions and correlated well with the number of amyloid deposits. According to the amyloid hypothesis, when soluble Aβ level (especially Aβ_42_, which is the species with the most propensity to aggregate) reaches a critical concentration, aggregation might begin to occur, leading to plaque formation. Therefore, the decrease in amyloid plaque formation, following chronic 5-HT_4_ receptor activation, might result from a shift of APP cleavage toward the non-amyloidogenic pathway. The apparent discrepancy with the absence of change in Aβ_42_ level following acute treatment with the 5-HT_4_ receptor agonist might reflect the slower kinetic of Aβ production, which requires two consecutive enzymatic cleavages. Further studies are needed to precisely characterize the effects of 5-HT_4_ receptor agonists on the release in CSF of other products of APP metabolism, an issue that we could not address in the present study due to insufficient amount of material available. Collectively, our results are consistent with a decrease in Aβ accumulation that consequently diminish the number of amyloid plaques in diseased brains and with a preventive action of chronic treatment with RS 67333.

### REDUCTION IN ASTROGLIOSIS AND MICROGLIOSIS

We demonstrated a clear decrease in glial reactivity upon chronic 5-HT_4_ receptor activation, in contrast with recent results using the 5-HT_4_ agonist SSP-002392 in APP/PS1 mice (Tesseur et al., 2013). The explanation for such discrepancy in the effect of two 5-HT_4_ receptor agonists requires to be elucidated. The 5-HT_4_ receptor is coupled to G_S_ protein, thus its activation by agonists induces cAMP production, an event linked to anti-inflammatory processes in the microglia (Kim et al., 2000). Moreover, 5-HT_4_ receptor agonists have been described to inhibit the interferon-γ-mediated immune response in cultured astrocytes (Zeinstra et al., 2006) and to have an anti-inflammatory role in the intestinal smooth muscle layer (Tsuchida et al., 2011). Therefore, 5-HT_4_ receptor stimulation is more likely to reduce inflammation. In addition, as amyloid deposits seem to induce inflammation in diseased brains (Akiyama et al., 2000), reducing Aβ burden and plaque number should concomitantly decrease inflammation. The new agonist SSP-002392 used in the previous study may have side effects that are non-related to its agonist action on 5-HT_4_ receptors, as suggested by Tesseur et al. (2013). Here, we antagonized the action of RS 67333 on plaque number and Aβ load by co-administration of a specific 5-HT_4_ receptor antagonist, RS 39604, that produced no effect when administered alone in 5XFAD mice. Both compounds have been reported to bind sigma receptors expressed in various brain regions (Eglen et al., 1995; Hegde et al., 1995), but no data are available on isolated σ_1_ receptor, the only sigma receptor cloned to date. Although it is unlikely that RS 67333 and RS 39604 act as an agonist and an antagonist at sigma receptors, respectively, using other 5-HT_4_ antagonists with different chemical structure, such as GR 113808 or GR 125487, would definitely exclude sigma receptor involvement. Inflammation progression is linked to AD course and long-term treatment with anti-inflammatory drugs reduces the risk to develop the disease. Reducing astrogliosis and microgliosis might thus have a beneficial outcome and contribute to slow down the pathology (Wyss-Coray and Rogers, 2012).

### EARLY INTERVENTION

In this study, we compared different protocols of chronic administration of RS 67333 to obtain the most efficient preventive effect in 5XFAD mice that were in the “prodromal stage” of disease (i.e., before the appearance of cognitive decline; **Figure 2A**). We observed the strongest effects with protocol 1, which combined very early treatment onset (1-month-old mice) and longest duration (3 months). In contrast, the 1-month treatment at a later stage of disease was insufficient to trigger significant improvement, consistent with the observation by Tesseur et al. (2013) who reported that a 37-day treatment was ineffective in APP/PS1 mice. 5-HT_4_ receptors undergo rapid and sustained desensitization in neurons (Ansanay et al., 1992) and, consequently, they could be down-regulated upon prolonged agonist exposure. To limit this risk, we used a partial 5-HT_4_ receptor agonist at moderate doses (1 mg/kg) and only twice a week, not daily. Finally, our findings demonstrate that a 2-month-long chronic treatment is already sufficient to prevent the appearance of cognitive deficits in 5XFAD mice. Collectively, our results clearly show that 5-HT_4_ agonists given at an early stage of the disease (before the appearance of cognitive decline) act preventively on Aβ formation and can preserve memory performance in 5XFAD mice. The necessity of early intervention regarding AD mouse models has also been established for other therapeutic strategies such as γ-secretase inhibition (Das et al., 2012).

In line with our results, it has been shown that enhancing serotonin signaling by administration of selective serotonin reuptake inhibitors (SSRI) is associated with lower Aβ levels and reduced number of plaques in mice and humans (Cirrito et al., 2011). Amyloid accumulation in the brain can precede AD symptoms by 10 years (Morris and Price, 2001) and even by more than 25 years in genetic forms of AD (Bateman et al., 2012). Therefore, acting as early as possible in the disease process seems to be crucial to slow down the pathology evolution. Although further investigations are needed to understand the complexity of 5-HT_4_ agonist actions, our data strongly suggest that these compounds are promising disease modifying-agents for AD.

## Conflict of Interest Statement

The authors declare that the research was conducted in the absence of any commercial or financial relationships that could be construed as a potential conflict of interest.
